# Coverage of Nutrition Interventions Intended for Infants and Young Children Varies Greatly across Programs: Results from Coverage Surveys in 5 Countries^1^,^2^,^3^

**DOI:** 10.3945/jn.116.245407

**Published:** 2017-04-12

**Authors:** Magali Leyvraz, Grant J Aaron, Alia Poonawala, Marti J van Liere, Dominic Schofield, Mark Myatt, Lynnette M Neufeld

**Affiliations:** 4 Global Alliance for Improved Nutrition, Geneva, Switzerland; and; 5 Brixton Health, Llawryglyn, United Kingdom

**Keywords:** coverage, fortified complementary foods, supplements, infant and young child nutrition, program monitoring, program evaluation, micronutrient powders

## Abstract

**Background:** The efficacy of a number of interventions that include fortified complementary foods (FCFs) or other products to improve infant and young child feeding (IYCF) is well established. Programs that provide such products free or at a subsidized price are implemented in many countries around the world. Demonstrating the impact at scale of these programs has been challenging, and rigorous information on coverage and utilization is lacking.

**Objective:** The objective of this article is to review key findings from 11 coverage surveys of IYCF programs distributing or selling FCFs or micronutrient powders in 5 countries.

**Methods:** Programs were implemented in Ghana, Cote d’Ivoire, India, Bangladesh, and Vietnam. Surveys were implemented at different stages of program implementation between 2013 and 2015. The Fortification Assessment Coverage Toolkit (FACT) was developed to assess 3 levels of coverage (message: awareness of the product; contact: use of the product ≥1 time; and effective: regular use aligned with program-specific goals), as well as barriers and factors that facilitate coverage. Analyses included the coverage estimates, as well as an assessment of equity of coverage between the poor and nonpoor, and between those with poor and adequate child feeding practices.

**Results:** Coverage varied greatly between countries and program models. Message coverage ranged from 29.0% to 99.7%, contact coverage from 22.6% to 94.4%, and effective coverage from 0.8% to 88.3%. Beyond creating awareness, programs that achieved high coverage were those with effective mechanisms in place to overcome barriers for both supply and demand.

**Conclusions:** Variability in coverage was likely due to the program design, delivery model, quality of implementation, and product type. Measuring program coverage and understanding its determinants is essential for program improvement and to estimate the potential for impact of programs at scale. Use of the FACT can help overcome this evidence gap.

## Introduction

The importance of adequate nutrition during early life for survival, growth, and development is well established (1, 2). Current guidelines include exclusive breastfeeding for the first 6 mo of life, with continued breastfeeding and timely introduction of safe and nutritionally adequate and developmentally appropriate complementary foods at month 6 (3). Complementary foods should be based on acceptable and available home foods, ensuring consistency and nutrient density through special preparation (1). The nutrient composition of complementary foods commonly consumed during the first 2 y of life in low- and middle-income countries has been well studied and found to be lacking in a number of nutrients in many contexts (4). When using linear programming and a series of assumptions related to affordability and acceptability, the use of local foods to increase quality and nutrient density can be improved. A recent review of such studies (5) showed that, even when the use of local ingredients could be optimized, it was difficult to meet requirements for some nutrients, such as iron and zinc, under economic and availability constraints. In such contexts, the WHO and others recommend that children should receive fortified complementary foods (FCFs)^6^ or nutrient supplements to address these dietary gaps (1). The efficacy (i.e., impact under controlled conditions) of such products to improve nutritional status and some functional outcomes is well established (6, 7). Many products are available on the market even in low- and middle-income countries, and programs that provide such products free or at a subsidized price are implemented at small and large scale in many countries around the world. A number of challenges in the quality of implementation of such programs, however, has been documented (8). Information on the coverage and utilization—and thereby potential for impact—of these programs is lacking.

The Global Alliance for Improved Nutrition worked with partners to implement a series of programs with the common objective of identifying, developing, and scaling up sustainable approaches to improve quality and access (availability and affordability) of FCFs and other nutritional products, such as micronutrient powders (MNPs), during the complementary feeding period. Through this grant, programs were implemented in 11 countries. Further details on some of these programs can be found elsewhere (9–11). Case studies provided important details on the process of engagement with governments, business, and organizations to identify delivery strategies and on the details of product improvement (9–11). From 2013 to 2015, the Global Alliance for Improved Nutrition conducted coverage surveys in 5 of the 11 countries to measure coverage and utilization of the products with the use of the Fortification Assessment Coverage Toolkit (FACT). This tool was designed specifically to assess coverage in both population-based and targeted fortification programs. The detailed results of some of the surveys have been published elsewhere (12–15). The objective of the analysis presented in this paper is to provide an overview of findings related to coverage and utilization across the 5 countries and discuss implications for future programming.

## Methods

### 

#### Program description.

An overview of the 5 country programs included in this compiled analysis is provided in **Table 1**. Briefly, the programs in Bangladesh and Vietnam distributed MNPs, the programs in Côte d’Ivoire and India distributed FCFs, and the program in Ghana distributed MNPs with added macronutrients. In Bangladesh, MNPs were sold door-to-door at a subsidized price by community volunteers who were paid on a commission basis. The program in the study region included additional training for the community volunteers on MNPs and sales bonuses when sales targets aligned with program use goals (60 sachets purchased for an individual child) were reached. The program in Vietnam was a 6-mo pilot program that focused on selling MNPs in >300 health centers across 4 provinces of the country (15). A behavior change communication (BCC) campaign and an intensive infant and young child feeding (IYCF) training of local health workers took place alongside delivery of the product. In Cote d’Ivoire, the program ran from 2011 to 2014 and supported a small-scale national producer to improve an FCF, aligned with recommendations on product composition and code-compliant labeling (14). The product was sold through retail shops in the capital city of Abidjan. A campaign was also developed and implemented by a nongovernmental organization to raise awareness and promote breastfeeding and complementary feeding practices, without promoting any specific product. In the State of Telangana, in India, a fortified complementary cereal product was distributed free of charge to children 6 mo to 5 y of age through the government-led Integrated Child Development Service program (13). No specific BCC activities or demand creation activities were implemented related to the product itself. In Ghana, a soy-based product similar to an MNP but with macronutrient content was distributed with the use of 2 different program models at pilot scale (16). Model 1 was implemented in the more rural northern regions. Petty traders sold the product door-to-door and at market stalls, and BCC and demand creation activities were delivered by community health workers at health care centers and in the community. In model 2, which focused on delivery in urban and periurban settings, the product was sold through small retail shops and roadside stalls. Demand creation through social marketing was implemented by a local social marketing firm.

**TABLE 1:** Overview of programs in 5 countries in which coverage surveys were implemented

Country	Product distribution	Product description	Product name	Launch date	BCC^1^ and demand creation activities
Bangladesh	Door-to-door sales by community health volunteers	Micronutrient powder	Pushtikona	2011	Distribution of posters, leaflets, calendars for self-monitoring of consumption; training of health workers and community leaders; cooking demonstrations in villages
Côte d’Ivoire	Sales through pharmacies and small retail shops	Fortified instant complementary food	Farinor and Nutribon	1998 and June 2011	Promotion of child feeding practices, including radio spots, materials, and community activities, including cooking demonstrations without reference to the specific commercial products
Ghana					
Model 1	Door-to-door sales by petty traders (part of a local microfinance initiative)	Micronutrient powder with additional macronutrients, lysine, and flavorings	Koko Plus	April 2013	Health extension workers delivered BCC and demand creation activities at primary health care centers and community events
Model 2	Sales through microretail routes (i.e., small shops, roadside stalls, and hawkers)	Micronutrient powder with additional macronutrients, lysine, and flavorings	Koko Plus	December 2012	Social marketing strategy implemented by local firm
India	Free distribution at government Integrated Child Development Service centers	Fortified complementary food	Bal Amrutham	June 2013	None specific to the program
Vietnam	Sales at community health centers	Micronutrient powder	Bibomix	June 2014	Training of health workers, visibility materials at the health center, free promotional items for mothers (bibs, bowls), and loudspeaker announcements

1 BCC, behavior change communication.

#### Survey procedures and sampling.

A total of 11 surveys in 5 countries (Bangladesh, Côte d’Ivoire, Ghana, India, and Vietnam) were conducted between February 2013 and April 2015 (**Table 2**). In Bangladesh, surveys were conducted shortly after program implementation and then 12 mo later; we also report here a single survey conducted shortly after implementation in a second region to which the program was expanded in 2015. In Côte d’Ivoire, a single survey was conducted in Abidjan at the close of the program. A total of 5 surveys were conducted in Ghana, 3 in model 1 and 2 in model 2. For model 1, the surveys occurred after 2, 10, and 14 mo of implementation. Door-to-door sales continued throughout this period, but the demand creation activities ceased at month 11 (i.e., 3 mo before the final survey). For model 2, surveys occurred 2 and 11 mo after program implementation began. In India, a single survey was conducted across the State of Telangana at the close of the program, 16 mo after initiation. In Vietnam, an end-line survey was conducted after a 6 mo pilot.

**TABLE 2:** Overview of sampling and methods used in 11 cross-sectional coverage surveys implemented in 5 countries

Country and phase or survey	Data collection	Survey area	Child age range, mo	Sample design^1^	Target sample size per survey, *n* child-caregiver pairs
Bangladesh^2^					
Survey 1.1A	September 2014	10 districts	6–59	2-stage spatial sampling (*m* = 16, *n* = 12)	1920
Survey 1.2A	August–September 2015	10 districts			1920
Survey 2A	March–April 2015	15 districts			2880
Côte d’Ivoire					
End-line	September–October 2014	9 communes in Abidjan	0–23	2-stage cluster sampling (*m* = 90, *n* = 13)	1142
Ghana^3^					
Survey 1.1B	July 2013	13 communities in northern Ghana	6–23	2 stage sampling of communities in intervention areas (*m* = 13, *n* = 24)	312
Survey 1.2B	May 2014	13 communities in northern Ghana			
Survey 1.3	September 2014	13 communities in northern Ghana			
Survey 2.1	February–August 2013	3 districts in eastern Ghana	0–23	Spatial sampling (*m* = 58, *n* = 18)	1044^4^
Survey 2.2	February–July 2014	3 districts in eastern Ghana			
India					
End-line	November–December 2014	State of Telangana	0–35	2-stage cluster random sampling (*m* = 90, *n* = 13)	1154
Vietnam					
End-line	November–December 2014	4 provinces	6–59	2-stage cluster random sampling (*m* = 8, *n* = 35)	1060

1 *m* = number of primary sampling units; *n* = number of caregiver-infant pairs per sampling unit.

2 Surveys took place at the end of a pilot phase before full roll-out (survey 1.1A) and 12 mo after roll-out (survey 1.2A) in the 10 phase-1 districts, and shortly after initiation in the 15 phase-2 districts (survey 2).

3 Surveys took place 2 mo (survey 1.1B), 10 mo (survey 1.2B), and 14 mo (survey 1.3) after project initiation in model 1, and 2 mo (survey 2.1) and 11 mo (survey 2.2) after project initiation in model 2. In model 1 districts, door-to-door sales continued, but demand creation activities stopped 3 mo before survey 1.3.

4 Coverage results presented for children >6 mo of age only (*n* = 640) per survey.

Survey procedures varied by country, but all used standardized survey instruments similar to those previously published to describe the surveys in Ghana (12), and to assess coverage of food fortification programs (17, 18). The survey instruments were adapted, translated, and pilot-tested in each setting to ensure that the language and wording of questions were clear, and that response options (e.g., food items used in the maternal dietary diversity question set) were appropriately adapted to context. Data were collected by trained interviewers under the supervision of experienced field supervisors. In 4 countries [Côte d’Ivoire, Ghana (model 2), India, and Vietnam], data were collected with the use of paper forms. In these surveys, data quality was ensured by supervision, interactive checking (for consistency and allowable values) during data entry, and batch checking (double-entry and validation, and batch application of consistency, range, and value checks) after data entry. In 2 countries [Bangladesh and Ghana (model 1)], data were collected with the use of mobile devices with only interactive checks for consistency, range, and permitted values used.

The target age range of the children included in the survey depended on the target population of each program. In Côte d’Ivoire and Ghana, the target age range was 6–23 mo of age, in India, the program focused on children 6–35 mo of age, and in Bangladesh and Vietnam, the program focused on those from 6 to 59 mo of age. All surveys were designed to be representative of the population in which the surveys took place.

#### Program coverage assessment.

Details on the research approach are described in the introduction (19) and article on large-scale food fortification coverage in this supplement (17). In brief, the FACT is a modular population-based survey instrument designed to assess coverage and quantify consumption of fortified foods. Stratified sampling is used to permit comparison of coverage estimates in populations known to have potential differences in access (e.g., urban compared with rural regions). In-depth sociodemographic and economic information, as well as other potential indicators of vulnerability, such as food security, permitted comparison of the results between those assumed to be at higher risk of inadequate dietary intake. The FACT was adapted to assess coverage of FCFs and nutrient supplements specifically designed for children 6 mo to 2, 3, or 5 y of age; the amount consumed and nutrient contribution from the products were not assessed.

In all surveys, data were collected on demographics and socioeconomic status; school attendance and education levels attained by household members; housing conditions; recent infant and child mortality; water, sanitation, and hygiene practices; food security; maternal dietary diversity; child health and nutritional status; IYCF practices; maternal and child anthropometric measurements; and coverage of the FCF or MNP intervention.

Where available, survey questions and resulting indicators were taken or adapted from validated instruments (19). The coverage indicator modules were adapted from the Semi-Quantitative Evaluation of Access and Coverage and Simplified Lot Quality Assurance Sampling Evaluation of Access and Coverage assessment tools (20), which were specifically designed to assess different levels of coverage (see indicator section below). As part of the coverage module, respondents were also asked to provide reasons for consumption and nonconsumption as a means of identifying potential barriers and factors that might facilitate coverage. The questions elicited unprompted responses related to motives for having given or not given the product to the child, and the responses were coded into categories; the exact wording of the questions varied by country and survey. Results are presented as response categories and indicate the surveys in which they were mentioned.

#### Ethical clearance and informed consent.

Ethical clearance to conduct the coverage surveys was obtained in each country from the institutional review board or ethics committee of the local institution involved in data collection (academic or government institution). Informed consent was obtained from the primary survey respondent on the basis that participation in the survey was voluntary. Oral consent was obtained in 3 countries (Côte d’Ivoire, Ghana, and India), and written consent was obtained in 2 countries (Bangladesh and Vietnam), as agreed upon with the corresponding institutional review board.

#### Indicators and data analysis.

Three levels of coverage were assessed for each survey, following the Tanahashi model of coverage (21). This model has proven useful for identifying major barriers to service delivery by separately assessing whether respondents have ever heard of the product (message coverage) and whether the product has ever been fed to the child (contact coverage). Finally, we assessed whether the child had been fed the product according to the pre-established program recommendation, i.e., adequate quantity with adequate frequency (effective coverage). In this manner, the exact interpretation of effective coverage in terms of the frequency of consumption of the product varied by country program, whereas the interpretation of message and contact coverage was the same across countries and surveys.

The potential to benefit from a nutritional product during the complementary period is dependent on whether those products fill nutrient gaps in the diets of young children, but dietary assessment was considered to be too complex for the resources (time and economic) available for these surveys. Instead we used 2 indicators of risk that have been found to be associated with inadequate dietary intake of nutrients in infants and young children: poverty and suboptimal IYCF practices. Poverty was determined with the use of a multidimensional poverty index (MPI) that includes nutrition and health, education, and living standards (12, 22). Unlike some wealth indexes that provide a ranking of relative poverty within a population, the MPI provides an objective estimate of poverty, and thus permits crosscountry comparisons of the extent of poverty in the survey sample. Suboptimal IYCF practices were determined with the use of the Infant and Child Feeding Index, which is an age-weighted score of breastfeeding, meal frequency, and dietary diversity (23, 24).

Each level of coverage is presented for each survey for the entire study population. The coverage ratio (CR) was then estimated as the ratio of coverage among those identified as being at risk to coverage among those identified as not being at risk. CRs in this type of population-based, prevention-focused program provide an opportunity to explore equity—specifically the extent to which the program is taken up by those presumed to be at higher risk of inadequate dietary intake based on previous literature, i.e., living in poverty and having children with suboptimal IYCF practices. A CR > 1 indicates that coverage is higher in those at risk than in those not at risk, thus suggesting that the product is more likely to reach those with the potential to benefit according to our study criteria. A CR < 1 indicates that the product has higher coverage among those not considered to be at risk according to our criteria. A CR = 1 indicates that the product coverage is equal in those considered to be at risk and those not considered to be so. The CR is presented separately for poverty and suboptimal IYCF.

Data were analyzed with the use of either R language for data analysis and graphics (version 3.2.2), scripts organized with the use of the R-Analytic Flow scientific workflow system (version 3.0.1), or SPSS (versions 16, 20, and 21). Summary statistics were calculated with the use of a blocked and weighted bootstrap estimator (14). A total of *r* = 400 bootstrap replicates were used. For each bootstrap replicate, a total of *m* primary sampling units (PSUs) were sampled with replacement (where *m* is the number of PSUs in the survey sample) from the survey data sets. PSUs were sampled with probability proportion to PSU population size with the use of a roulette wheel algorithm. Observations within selected PSUs were also sampled with replacement with the use of the same within-PSU sample size that was achieved in the survey.

## Results

### 

#### Characteristics of survey samples.

As expected by design, the sample populations are older in Bangladesh and Vietnam (mean age ∼30 mo) than in the other countries (mean age ranging from 11 to 17 mo) (**Table 3**). Poverty was high in the surveys in Bangladesh and the program areas of model 1 in Ghana, with ∼60–75% of the sample populations being classified as poor. A very small proportion of the populations in Vietnam were poor (7.3%), with India, Cote d’Ivoire, and the Ghana model 2 areas in between, ranging from 14.4% to 23.2% poor. Despite the poverty differences, from one-half to three-quarters of the children had suboptimal ICYF practices across all countries.

**TABLE 3:** Characteristics of households and children in 11 cross-sectional coverage survey samples from 5 countries^1^

Country and program stage	Sample size, *n*	Household size, *n*	Age, *n*	Poverty,^2^ %	Suboptimal IYCF,^3^ %
Bangladesh^4^					
Survey 1.1A	1927	5.0 (5.0, 5.2)	30 [6–59]	60.9 (57.6, 64.0)	66.2 (63.1, 69.3)
Survey 1.2A	1924	5.1 (5.0, 5.2)	31 [6–59]	70.4 (67.2, 73.6)	53.9 (50.8, 56.9)
Survey 2	2887	5.4 (5.3, 5.4)	30 [6–59]	61.5 (58.8, 64.3)	59.3 (56.9, 61.9)
Côte d’Ivoire					
End-line	1113	6.1 (5.8, 6.4)	11 [0–23]	21.0 (16.6, 26.3)	74.6 (69.9, 78.8)
Ghana^5^					
Survey 1.1B	306	NA	14 [6–23]	74.7 (62.1, 85.3)	51.6 (41.3, 61.8)
Survey 1.2B	306	NA	16 [6–24]	67.9 (53.6, 82.8)	56.1 (45.2, 68.2)
Survey 1.3	307	NA	14 [6–23]	58.3 (49.0, 67.8)	60.3 (52.6, 67.0)
Survey 2.1	620	NA	14 [6–24]	17.6 (13.6, 21.6)	70.4 (65.7, 74.8)
Survey 2.2	663	NA	15 [6–24]	14.4 (10.5, 17.9)	76.9 (72.7, 81.2)
India					
End-line	905	5.0 (4.9, 5.1)	17 [0–35]	23.2 (17.6, 29.9)	70.8 (65.5, 75.6)
Vietnam					
End-line	962	4.7 (3.8, 5.53)	28 [6–60]	7.3 (4.8, 10.2)	71.9 (65.4, 76.6)

1 Values are means (95% CIs) or means [ranges]. ICFI, Infant and Child Feeding Index; IYCF, infant and young child feeding; MPI, multidimensional poverty index; NA, not assessed.

2 Estimated with the use of the MPI and defined as MPI ≥0.33.

3 Classified with the use of the ICFI. Suboptimal was defined as having an ICFI score <6.

4 Surveys took place at the end of a pilot phase before full roll-out (survey 1.1A) and 12 mo after roll-out (survey 1.2A) in the 10 phase-1 districts and shortly after initiation in the 15 phase-2 districts (survey 2).

5 Surveys took place 2 mo (survey 1.1B), 10 mo (survey 1.2B), and 14 mo (survey 1.3) after project initiation in model 1, and 2 mo (survey 2.1) and 11 mo (survey 2.2) after project initiation in model 2. In model 1 districts, door-to-door sales continued, but demand creation activities stopped 3 mo before survey 1.3.

#### Coverage and equity in coverage by risk category.

Message coverage indicates that awareness of the product was low in Vietnam (<30%), moderate in Bangladesh (40–65% in the different surveys), and very high in Cote d’Ivoire, India, and Ghana (≥85% with the exception of one survey in Ghana) (**Table 4**). In general, this was reflected in high contact and effective coverage in model 1 in Ghana and in India. In model 2 in Ghana, message coverage reached >50% by the final survey, but effective coverage remained low and actually decreased. In Bangladesh and Cote d’Ivoire, contact coverage ranged from ∼20% to ∼40%, but effective coverage was extremely low (<5%). Interestingly, in Vietnam, despite overall low coverage at all levels, the progression among levels of coverage [from awareness (message = 29.0%) to use ≥1 time (contact: 22.6%) to use according to program recommendation (effective: 12.5%)] was less dramatic than it was in other programs. The detailed results of the surveys in Cote d’Ivoire, India, Ghana, and Vietnam have been published elsewhere (12–15).

**TABLE 4:** Message, contact, and effective coverage of the nutritional product during each survey^1^

Country and program stage	Sample size, *n*	Message coverage^2^	Contact coverage^3^	Effective coverage^4^
Bangladesh^5^				
Survey 1.1A	1927	44.7 (41.3, 48.4)	23.5 (20.8, 26.7)	2.1 (1.4, 3.2)
Survey 1.2A	1924	63.7 (60.0, 67.1)	36.8 (33.4, 40.9)	3.9 (2.7, 5.5)
Survey 2	2887	46.3 (43.3, 49.4)	26.3 (23.6, 28.6)	0.8 (0.4, 1.2)
Côte d’Ivoire				
End-line	776	85.0 (82.3, 87.3)	37.5 (32.8, 42.5)	4.6 (2.9, 7.2)
Ghana^6^				
Survey 1.1B	306	97.7 (92.9, 100.0)	94.4 (89.7, 98.1)	88.3 (81.1, 94.6)
Survey 1.2B	306	99.0 (91.8, 100.0)	92.0 (82.7, 98.7)	83.1 (73.4, 93.1)
Survey 1.3	307	99.7 (98.4, 100.0)	84.4 (77.6, 89.9)	61.9 (53.2, 69.9)
Survey 2.1	620	63.8 (57.2, 71.1)	23.5 (19.0, 28.5)	15.3 (11.3, 19.8)
Survey 2.2	663	89.8 (86.6, 92.4)	52.8 (47.7, 58.9)	9.4 (6.7, 12.4)
India				
End-line	905	93.7 (82.4, 97.9)	86.8 (73.1, 94.1)	57.2 (48.2, 65.8)
Vietnam				
End-line	962	29.0 (21.9, 35.9)	22.6 (17.4, 28.2)	12.5 (8.3, 16.8)

1 Values are % (95% CI).

2 Defined as ever having heard of the product.

3 Defined as ever having tried the product.

4 Defined as using the product at the frequency and quantity recommended by each individual program.

5 Surveys took place at the end of a pilot phase before full roll-out (survey 1.1A) and 12 mo after roll-out (survey 1.2A) in the 10 phase-1 districts and shortly after initiation in the 15 phase-2 districts (survey 2).

6 Surveys took place 2 mo (survey 1.1B), 10 mo (survey 1.2B), and 14 mo (survey 1.3) after project initiation in model 1, and 2 mo (survey 2.1) and 11 mo (survey 2.2) after project initiation in model 2. In model 1 districts, door-to-door sales continued, but demand creation activities stopped 3 mo before survey 1.3.

Generally, coverage did not differ significantly between those at risk and those not at risk (CI includes 1), with a few exceptions (**Table 5**). Most notably, message coverage was consistently higher among those with better IYCF practices in Bangladesh; the few other significant differences also showed higher coverage among those less at risk (CR < 1).

**TABLE 5:** CRs of message, contact, and effective coverage of the nutritional product during each survey by poverty and adequacy of infant and child feeding practices^1^

	Message coverage^2^		Contact coverage^3^		Effective coverage^4^	
Country and program stage	Poverty^5^	Suboptimal IYCF^6^	Poverty^5^	Suboptimal IYCF^6^	Poverty^5^	Suboptimal IYCF^6^
Bangladesh^7^						
Survey 1.1A	1.12 (0.98, 1.30)	0.81 (0.69, 0.93)*	1.12 (0.90, 1.41)	0.75 (0.59, 0.93)*	1.86 (0.67, 7.51)	0.26 (0.09, 2.34)
Survey 1.2A	1.03 (0.92, 1.14)	0.83 (0.75, 0.93)*	0.97 (0.82, 1.16)	0.76 (0.63, 0.92)*	0.78 (0.38, 1.97)	0.43 (0.20, 0.99)*
Survey 2	1.01 (0.89, 1.12)	0.86 (0.77, 0.97)*	0.98 (0.82, 1.15)	0.87 (0.73, 1.04)	0.32 (0.06, 1.06)	0.49 (0.12, 1.54)
Côte d’Ivoire						
End-line	0.88 (0.76, 0.99)*	1.02 (0.92, 1.14)	0.70 (0.47, 0.96)*	1.35 (0.99, 1.88)	0.71 (0.09, 2.15)	1.83 (0.61, 8.86)
Ghana^8^						
Survey 1.1B	1.00 (0.95, 1.04)	1.01 (0.98, 1.08)	0.98 (0.91, 1.06)	0.97 (0.91, 1.06)	0.90 (0.80, 1.03)	0.91 (0.79, 1.06)
Survey 1.2B	1.00 (0.90, 1.05)	1.02 (0.99, 1.12)	0.93 (0.80, 1.04)	1.09 (0.98, 1.28)	1.09 (0.88, 1.42)	1.11 (0.97, 1.32)
Survey 1.3	1.01 (0.99, 1.05)	0.98 (0.97, 1.00)	0.95 (0.84, 1.10)	0.90 (0.77, 1.04)	1.00 (0.77, 1.27)	0.97 (0.76, 1.23)
Survey 2.1	1.07 (0.88, 1.25)	0.99 (0.85, 1.18)	0.99 (0.62, 1.43)	0.67 (0.44, 0.99)*	1.35 (0.70, 2.22)	0.81 (0.52, 1.33)
Survey 2.2	0.99 (0.90, 1.07)	1.05 (0.98, 1.14)	0.85 (0.65, 1.07)	1.22 (0.96, 1.60)	1.42 (0.71, 2.67)	0.93 (0.53, 2.03)
India						
End-line	0.94 (0.83, 1.01)	0.98 (0.95, 1.02)	0.96 (0.82, 1.06)	0.99 (0.92, 1.07)	0.91 (0.71, 1.14)	0.95 (0.79, 1.14)
Vietnam						
End-line	0.74 (0.34, 1.30)	0.99 (0.73, 1.38)	0.84 (0.36, 1.53)	1.14 (0.77. 1.81)	0.76 (0.10, 1.98)	1.06 (0.64, 1.82)

1 Values are ratios (95% CIs). CR is the ratio of coverage between those defined as at risk (poverty, suboptimal IYCF practices) and those defined as not at risk (no poverty, adequate IYCF practices). CR > 1 implies that coverage is higher in those at risk than in those not at risk; CR < 1 implies that coverage is lower in those not at risk than in those at risk. *Statistically significant CR, *P* < 0.05. CR, coverage ratio; ICFI, infant and child feeding index; IYCF, infant and young child feeding; MPI, multidimensional poverty index.

2 Defined as ever having heard of the product.

3 Defined as ever having tried the product.

4 Defined as using the product at the frequency and quantity recommended by each individual program.

5 Estimated with the use of the MPI and defined as MPI ≥0.33.

6 Classified with the use of the ICFI. Suboptimal was defined as an ICFI score <6.

7 Surveys took place at the end of a pilot phase before full roll-out (survey 1.1A) and 12 mo after roll-out (survey 1.2A) in the 10 phase-1 districts and shortly after initiation in the 15 phase-2 districts (survey 2).

8 Surveys took place 2 mo (survey 1.1B), 10 mo (survey 1.2B), and 14 mo (survey 1.3) after project initiation in model 1, and 2 mo (survey 2.1) and 11 mo (survey 2.2) after project initiation in model 2. In model 1 districts, door-to-door sales continued, but demand creation activities stopped 3 mo before survey 1.3.

#### Motives for use and nonuse of FCFs and nutritional supplements.

There was considerable variability in the reported motives for use and nonuse of FCFs across countries. In 3 countries, reasons for nonuse of the products included irregular or insufficient supply or availability of product, perceived undesirable side effects of the product, and lack of behavior communication or demand creation activities (**Table 6**). In 4 countries, respondents mentioned perceived benefits of the product as being a motivator for product use (**Table 7**).

**TABLE 6:** Factors identified by survey respondents as barriers to product use (in order by descending frequency)^1^

Barriers	Bangladesh	Côte d’Ivoire	Ghana—model 1	Ghana—model 2	India	Vietnam	Total count
Irregular or insufficient supply	●	○	○	○	●	●	3
Perceived side effects (i.e., diarrhea and vomiting)	●	○	○	○	●	●	3
Interrupted or nonexistent behavior change communication or demand creation activities	○	●	●	○	●	○	3
Incorrect preparation or use	●	○	○	○	○	●	2
Intrahousehold sharing	○	●	○	○	●	○	2
Cost of product, lack of purchase power^2^	●	○	○	●	○	○	2
Disliking the product’s taste, flavor, or color	●	○	○	○	○	●	2
Distance to the point of distribution	○	○	○	○	●	●	2
Lack of visible improvement in child’s health	○	○	○	○	○	●	1
Lack of awareness of product	○	○	●	○	○	○	1
Perceived lack of need	●	○	○	○	○	○	1
Poor general infant and young child feeding practices	○	●	○	○	○	○	1
Program duration too short	○	○	○	●	○	○	1
Husband or family refusal	○	○	○	○	●	○	1

1 The question elicited responses from survey participants to identify (unprompted) factors that had motivated them to give the product to their child; the exact wording of the question varied by survey and country. ● = mentioned by respondents in that country; ○ = not mentioned by respondents in that country.

2 India was the only country in which the product was provided free of charge.

**TABLE 7:** Factors identified by survey respondents favoring product use (in order by descending frequency)^1^

Boosters	Bangladesh	Côte d’Ivoire	Ghana—model 1	Ghana—model 2	India	Vietnam	Total count
Positive perception of the product (i.e., healthy, improved appetite, reduced micronutrient deficiencies)	●	●	○	○	●	●	4
High awareness, acceptability, and use of similar products before program	○	●	●	○	○	○	2
Simultaneous and intensive behavior change communication and demand creation	○	○	●	○	○	●	2
Trust in brand or source	○	●	○	○	○	●	2
High awareness and use of the government program	○	○	○	○	●	●	2
Liking the product’s taste	○	○	○	○	●	○	1
Product was free^2^	○	○	○	○	●	○	1
Availability of different package sizes and prices	○	○	○	○	○	●	1

1 The question elicited responses from survey participants to identify (unprompted) factors that had motivated them to give the product to their child; the exact wording of the question varied by survey and country. ● = mentioned by respondents in that country; ○ = not mentioned by respondents in that country.

2 India was the only country in which the product was provided free of charge.

## Discussion

The need for more frequent and better assessments of nutrition program coverage and better understanding of the pattern of use of nutritional products, particularly during the complementary feeding period, has been well established (25, 26). Yet tools to support the standardized collection of such information have been lacking. In this paper, we report the application of the FACT in 5 countries in which programs focused on increasing accessibility and, ultimately, coverage and utilization of FCFs or other nutritional supplements intended for young children through a variety of delivery models. Surveys found wide variability in coverage, which was not unexpected, given the variable program designs and durations.

The variability in coverage likely is due at least in part to the differences in delivery platforms, which may also explain the different patterns of progression from message to contact to effective coverage. For example, in Vietnam, only one-third of the sample surveyed had heard of the product (message coverage), but the majority of those surveyed had used it ≥1 time (contact coverage), and many even used it regularly (effective coverage). This may suggest that the delivery platform (health centers) was effective in communicating, creating demand for the product, and overcoming other barriers, such as availability and acceptance, among others. Whether the low message coverage was related to low use of the health centers generally or lack of focus on the program within some health centers was not documented in the survey but would be critical to determine if the program was to be further scaled. In India, high use of the Integrated Child Development Service systems likely facilitated the high message and contact coverage, but the lack of an accompanying communication strategy may have limited effective coverage.

Of all surveys included in this analysis, high effective coverage (>80%) was achieved only in the first of the 2 program models implemented in Ghana, i.e., the door-to-door sales model. This would suggest that the pricing was affordable to the population and that the strategy to raise awareness and create demand was highly effective. The convenience factor of door-to-door sales may have also favored coverage, but the drop in effective coverage 3 mo after the demand creation activities ended (Table 4; survey 1.3) highlights the importance of demand creation, even when convenience has been addressed. The high coverage in Ghana may also be related to the fact that the program was implemented at small scale, with continual feedback from the coverage surveys, which may have facilitated continual course correction in diverse program activities. Details of the content of that program and communication campaign have been published elsewhere (12, 16). On the contrary, in Bangladesh—also a sales model through home visits by community sales agents—despite the fact that it created awareness in more than one-half of the population (i.e., message coverage), other barriers likely limited contact, and particularly effective coverage. Challenges related to the frequency and regularity of home visits by the sales agents, the quality of their training, and the regularity of supply, among others, have now been clearly identified in subsequent studies and are being used to improve the quality of implementation (27, 28).

As shown in Côte d’Ivoire and model 2 in Ghana, the retail market model was highly effective at creating awareness of the product, but this did not translate into high contact and effective coverage. This may be due to several factors, including lack of effective demand creation activities and the short duration of the programs. For example, successful commercialization of new products may take ≤6–8 y, according to industry benchmarks (29). One of the limitations of the surveys was the lack of data collected for the full range of complementary feeding products available, and thus the inability to compare coverage and utilization of other commercially available complementary foods or nutritional supplements.

Although the information related to potential barriers and factors facilitating coverage is limited in all the surveys reported here, we can conclude that even when supply issues are addressed, awareness of the product alone is insufficient to achieve high coverage and create demand. Essential components to effectively change behaviors related to IYCF were extensively studied in the Alive and Thrive program in Bangladesh, Ethiopia, and Vietnam (30). These evaluations stressed the importance of a multicomponent, multichannel communications strategy that includes community mobilization (i.e., raising awareness of the issues and gaining buy-in for the needed behaviors from all members of society, not just the caregiver), sustained mass media campaigns to further raise awareness, and regular and quality interpersonal communication to motivate and address individual barriers (30).

The type of product itself, FCF or nutritional supplement, may also be one factor influencing coverage. FCFs are widely known in many contexts and are already present on the market in most countries, and therefore the need to create awareness may be lower than with MNPs or similar products that are new to caregivers. Intrahousehold sharing was a common barrier to achieving high effective coverage with FCFs, a tendency that has been well documented previously (31, 32) and something that has proven extremely difficult to modify in programmatic settings (33). Intrahousehold sharing may be less of a concern with products not perceived as foods. For example, in one study in Mexico, MNPs were perceived as vitamin and mineral supplements rather than foods, and therefore were considered appropriate for targeting to individuals within the household (34). However, this raised other challenges, because they were perceived as appropriate for short-term use and to alleviate specific deficiencies, but not as something to be provided for extended periods of time (34). Another potential influence on awareness, acceptance, and use of the programs generally and the nutritional products specifically is the capacity and enthusiasm of program staff, a factor that we were unable to assess in these surveys.

One of the objectives of these surveys was to determine whether there were differences in coverage between those more or less vulnerable, defined as below a poverty cutoff (per MPI) or according to IYCF practices. In most surveys, there was no clear pattern between coverage and vulnerability, with 2 notable exceptions. In Côte d’Ivoire, the product appeared to have greater visibility and use among the nonpoor. The consistency of the CR between message, contact, and effective coverage (not significantly different but in the similar direction) might suggest that information related to the product was less likely to reach the poor. Price was not identified as a barrier to use by respondents in this survey, but that may also reflect that the poor were less aware of the product’s existence. There was considerable civil strife in Cote d’Ivoire over the course of the program, and whether this influenced awareness raising, supply, or people’s ability or willingness to purchase the product cannot be ascertained from this survey. In Bangladesh, there was a consistent tendency toward lower awareness and use of MNPs in those with suboptimal IYCF practices (i.e., CR < 1), but not in those classified as poor (CR not different than 1). Whether this reflects self-selection of the sales agents, e.g., by their visiting the women in their community who they believed were most likely to accept and purchase the product, cannot be determined from these surveys. The importance of the education of mothers in general and caregivers’ knowledge and understanding of IYCF in the willingness to use complementary feeding products has been shown elsewhere (35, 36). This suggests that education campaigns may need to develop specific modules to overcome knowledge and use challenges for caregivers with lower levels of education in general or with limited knowledge of IYCF. Similarly, such issues should be addressed as part of the training for the sales agents to ensure that they have capacity to identify and address such challenges.

There are several strengths to these surveys that should be highlighted. The survey designs allowed for comparisons of coverage between more and less vulnerable groups, which, to our knowledge, is a unique contribution to the literature and something that we believe should be standard practice to understand the potential for impact of programs among those presumably most likely to benefit. The use of standardized and validated questionnaires in the FACT modules permitted the comparison of results across surveys in very different country and programmatic contexts. In particular, the standard definition of message and contact coverage allowed for unique crossprogram and crosscountry comparisons. One limitation is that, given variable program design and goals, effective coverage cannot be directly compared in a similar manner. Our reliance on caregiver recall to assess use may introduce bias toward what would be considered socially acceptable responses. This is a common challenge with any survey of product use, and we minimized this potential by implementing survey activities separately from program activities.

## Conclusions

Many nutrition interventions to improve infant and young child nutrition have proven to be efficacious in controlled trials, but understanding how to translate this into impactful programs at scale has been challenging. Important gaps in understanding the nature of these challenges exist, including a dearth of information related to program coverage and utilization of IYCF products distributed or sold as a part of those programs or made available through commercial channels. Measuring and understanding determinants of program performance, including coverage, throughout program implementation is essential for program improvement and to estimate the potential for impact. Whether targeted or not, programs that distribute or facilitate the sale of nutritious products should be clear at design who they intend to reach, know the dietary gaps they intend to fill, set time-bound targets related to the overall program goals, then generate information as part of monitoring and/or evaluation activities to measure performance against these targets. Although obvious, such goals and targets are not consistently set in programs, and assessment of program performance is often limited. Use of the FACT as part of evaluation activities developed with standardized methodologies and validated indicators can help overcome the gaps in the collection of such information. The findings of the series of surveys presented here show that coverage of population-based interventions intended for infants and young children varies greatly depending on design and delivery model. Achieving impact at scale of such programs will be feasible only if patterns and determinants of coverage and utilization are assessed and the resulting evidence is used to inform improvements in design and implementation.

## Footnotes

6 Abbreviations used: BCC, behavior change communication; CR, coverage ratio; FACT, Fortification Assessment Coverage Toolkit; FCF, fortified complementary food; IYCF, infant and young child feeding; MNP, micronutrient powder; MPI, multidimensional poverty index; PSU, primary sampling unit.
